# Morphological Measurements of Innominate Foramina and Bony Spurs along the Base of Sphenoid as a Potential Risk Factor for Neurovascular Entrapment, Radiological Interpretation and Surgical Access

**DOI:** 10.21315/mjms2023.30.2.8

**Published:** 2023-04-18

**Authors:** Roshni Sadashiv, Suresh Managutti, Veena Kulkarni, Arun V Kulkarni, Umesh Dixit

**Affiliations:** 1 Department of Anatomy, SDM College of Medical Sciences and Hospital, Shri Dharmasthala Manjunatheshwara University, Dharwad, India; 2 Department of Community Medicine, SDM College of Medical Sciences and Hospital, Shri Dharmasthala Manjunatheshwara University, Dharwad, India

**Keywords:** foramen innominate, skull base, infratemporal fossa, bony bar, sphenoid

## Abstract

**Background:**

Restricted access and compression of neurovascular structures at various anatomic variations at the skull base poses a challenge to surgeons, neurologists and anesthetists. The present study was performed with the objective of providing morphometric analysis of innominate foramina, and anomalous bony bars and spurs along the infratemporal surface of the greater wing of the sphenoid and reviewing the practical significance of dealing with this region.

**Methods:**

A total of 100 dry-aged human adult skulls from the archives of the osteology library of the Department of Anatomy were studied. A detailed morphometric analysis of such innominate foramina and anomalous osseous structures along the base of the sphenoid was performed using a sliding digital vernier caliper.

**Results:**

Anomalous bony bar was found in 22 skulls (25.28%). A complete bar was observed at eight (9.1%). An innominate foramen was located inferomedial to foramen ovale (5 unilateral and 3 bilateral) with a mean anteroposterior diameter of 3.44 mm and a mean transverse diameter of 3.16 mm.

**Conclusion:**

Neurovascular structures may be compressed by abnormal bony outgrowths or while traversing through such unnamed bony foramina. The latter may also be overlooked and mistaken during radiological interpretation leading to delayed diagnosis. Such unnamed foramina and bony outgrowths need to be documented in the literature due to their surgical, and radiological implications and limited citations.

## Introduction

The region of the base of the skull poses a challenge to clinical anatomists and surgeons as they encounter various foramina being bridged by either ligaments or bony bars. This is more common in the region of the sphenoid bone (1). Foramen ovale and foramen spinosum is routinely present foramina along the posterior part of the greater wing of the sphenoid at the transit zone between the middle cranial fossa and infratemporal fossa. Occasionally, one can find the Canaliculus Innominate of Arnold between the former two foramina transmitting lesser petrosal nerve (2). Ossification of intrinsic ligaments of sphenoids such as the inter clinoid, carotidoclinoid, pterygospinous and pterygoalar ligaments are well documented in the literature (3). Pterygospinous and pterygoalar ligaments are often described in the literature as syndesmotic ligaments stretching from the posterior free border of the lateral pterygoid plate to the spine of the sphenoid or infratemporal surface of sphenoid bone respectively (4). Complete ossification or calcification of these ligaments leads to the formation of bony bars, pterygospinous and pterygoalar foramen (porus crotaphitico-buccinators) in close proximity with foramen ovale (4, 5). The cause of the ossification of ligaments at the cranial base is not fully understood. However, some are of the opinion that it could be due to chemical, genetic factors or an age-dependent process (4–6).

The skull base can present with numerous small foramina that could be emissary foramen mistaken for an abnormality or formed due to the ossified bony ligaments (7). The bony bars can inadvertently affect the nerves and vessels traversing various innominate foramina via the foramen ovale or foramen spinosum. This can result in neurovascular disturbances along their areas of distribution. Conversely, these accessory osseous structures need not always be present in clinical manifestations and could largely depend on the location and extent of injury to adjoining anatomical entities (8). The current study describes a morphometric analysis of such innominate foramina, anomalous osseous structures along the base of sphenoid and discusses its relevance in terms of clinical applications.

## Methods

A total of 100 dry aged human adult skulls available with their records of age and origin from a collection of contemporary skulls belonging to the osteology library of the department of Anatomy were studied. Broken, fractured and skulls with pathological deformities were excluded from the study. The skull base was macroscopically inspected for innominate foramina and anomalous bony bars by two anatomists. The anterior-posterior and transverse diameters of the foramen were measured using a sliding digital vernier calliper. The structures of interest were photographed using a high-resolution digital camera (9).

## Results

**Figure 1:**
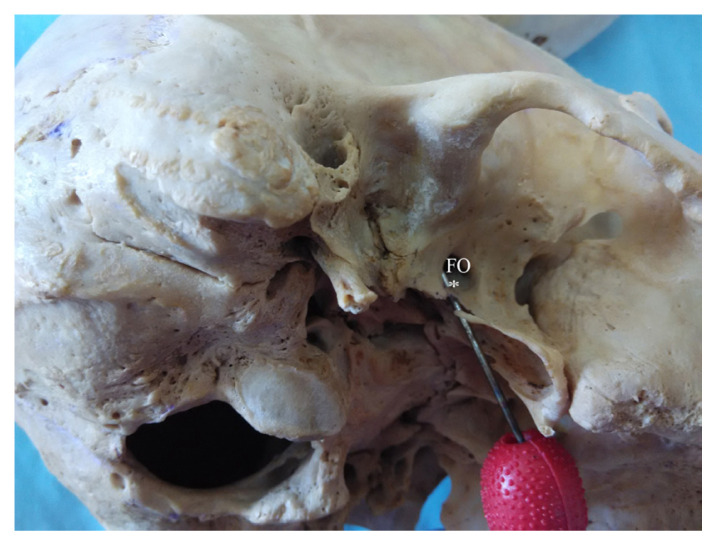
Lateral right view of the infratemporal region of the skull. An innominate foramen (* probed) is seen located inferomedial to and communicating with the foramen ovale (FO)

**Figure 2:**
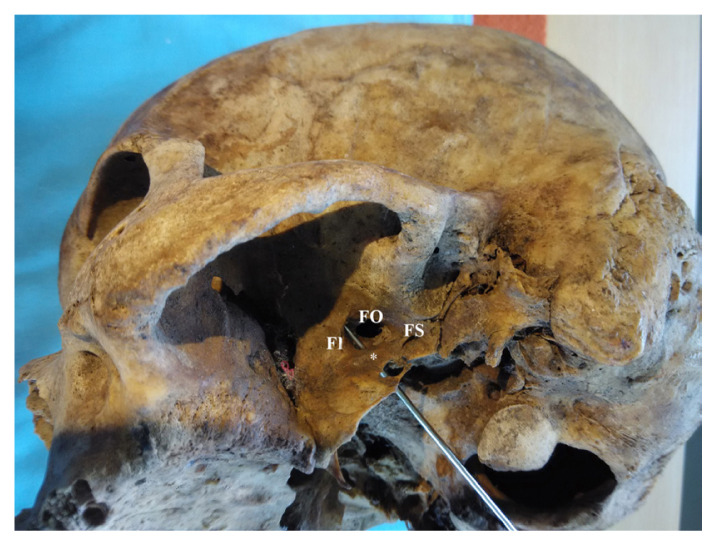
Lateral left view of the infratemporal region of the skull. A thick bony bar * bridging across the root of the lateral pterygoid plate (LP) and anterolateral to foramen spinosum (FS) resulting in foramen innominate (FI) (probed foramen)

**Figure 3:**
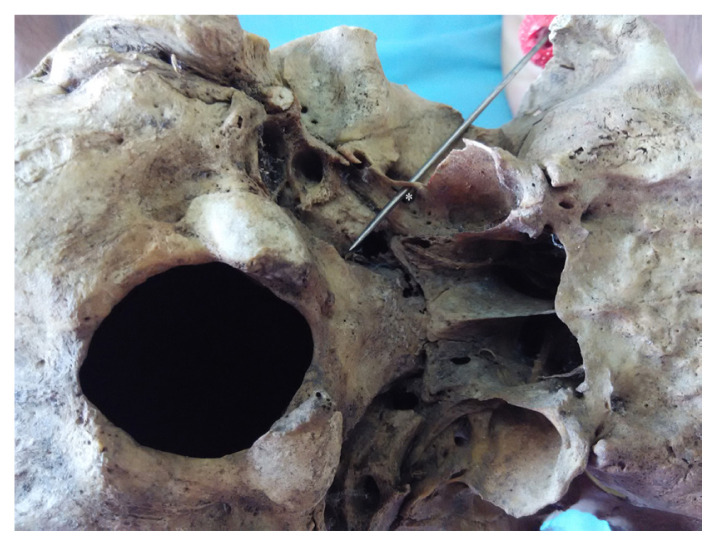
Skull base as seen above. A thin bony bar * bridging across the root of the lateral pterygoid plate and anterolateral to foramen spinosum resulting in foramen innominate (probed foramen)

**Figure 4:**
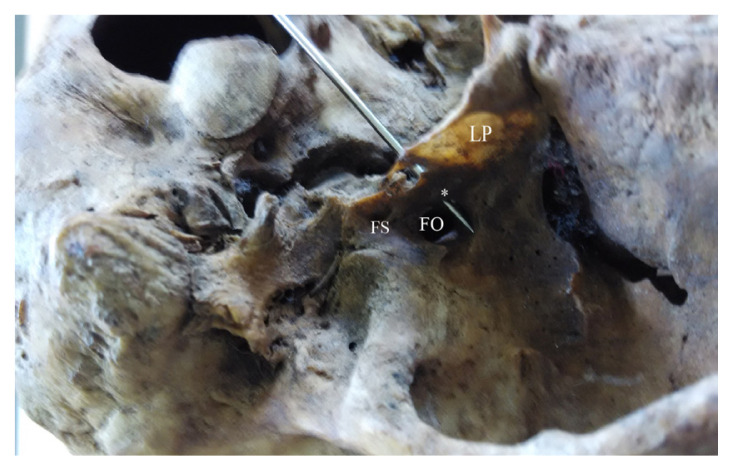
Skull base as seen above. A narrow innominate foramen (* probed) was seen medial to foramen ovale (FO) and foramen spinosum (FS), communicating with FO. Note: LP-lateral pterygoid plate

In this study, we investigated 100 dry human adult skulls and detected an anomalous bony bridge extending from the root of the lateral pterygoid plate towards the anteromedial or anterolateral edge of foramen spinosum. An innominate foramen medial to and communicating with the foramen ovale was observed. Anomalous bony bars were found in 22 skulls (25.28%) of the total sample of 100 skulls examined (Figures 1–4). Fourteen skulls (16.09%) showed partial bony projections from the root of the lateral pterygoid and from the anteromedial or anterolateral edge of foramen spinosum. The mean distance between these bony projections was 6.79 mm (SD = 2.74 mm). As illustrated in Figure 3, a complete bar was observed in eight specimens (9.1 %) with a mean length of 5.36 mm (SD = 2.29 mm). An innominate foramen thus formed was located inferomedial to foramen ovale (5 unilateral and 3 bilateral) as presented in Figures 1 and 2. The shape of the foramen varied from oval to circular with a mean anteroposterior diameter of 3.44 mm (SD = 0.47 mm) and a mean transverse diameter of 3.16 mm (SD = 0.56 mm). The mean distance of the innominate foramen from the nearest bony landmark such as foramen ovale and foramen spinosum was measured (Table 1).

## Discussion

### Neurovascular Compression due to Abnormal Bony Growth at the Skull Base

Infratemporal fossa is an anatomical space located below the middle cranial fossa. It comprises dense neurovascular structures, and muscles of mastication arising from the pterygoid plates, temporalis, and the Eustachian tube (10). Neurovascular compression of the cranial nerve as it exits the brain stem through a constellation of foramina is responsible for various symptoms of neuralgia (11). In the case of trigeminal neuralgia, the most commonly affected nerve division is the mandibular nerve, with the ophthalmic division being the least affected (11). In 20% of cases, mandibular neuralgia is caused due to compression by abnormal bony outgrowths at the skull base (12). The mandibular nerve passes through the foramen ovale into the infratemporal fossa and its branches supply the derivatives of the first pharyngeal arch. The ossification of intrinsic ligaments or bony bridges in relation to foramen ovale may be responsible for a series of symptoms such as weakness in muscles of mastication, numbness of buccal region, functional impairment of salivation, taste loss in anterior rd of the tongue, following compression of motor or sensory branches of mandibular nerve (13). The mandibular nerve and its branches can be compressed between the muscle and the lateral pterygoid plate. The presence of such osseous bars also poses a restriction to trans ovate and intracisternal injections for trigeminal neuralgia (14). Schwannomas, tumours such as inverted papilloma, juvenile angiofibroma, adenoid cystic carcinoma and squamous cell carcinoma are other lesions involving the region of the infratemporal fossa. The surgical approach to this area is a key task despite significant advances in optics, instrumentation and endonasal techniques. Thus, identifying these mineralised bony ligaments leading to innominate foramina and, bony outgrowth would aid in planning surgical procedures for easy access and mobilisation (15).

In the present study, a bony bridge extending from the root of the lateral pterygoid plate towards the anteromedial or anterolateral edge of foramen spinosum was identified. Bony spines were protruding from the latter in 17% of cases. They were clearly different from the pterygoalar and pterygospinous bony bars. The foramen innominate thus formed was communicating with the foramen ovale. It had no communication with the middle cranial fossa, ruling out the possibility of a common variant of foramen rotundum with an average diameter of 1 mm–3 mm that usually opens between pterygoid plates of the sphenoid (7). The bony bars forming the foramen did not divide the foramen ovale; rather they formed an innominate foramen that communicated with the latter. On close visual inspection of the foramen, there were no bony spurs extending from the rim of their lumen. In the majority of cases, the bony bar was present medial to foramen ovale and foramen spinosum. Nevertheless, the shape, size and diameter of the foramen (3.44 mm × 3.16 mm) seem to have been large enough to allow normal passage of the nerve. Probably the neurovascular structures are vulnerable to compression on their passage through foramen innominate of smaller diameter. It was hard to determine whether the formation of such foramina is developmental or secondary to the ossification of unnamed ligaments and bony bars. Given the paucity of literature and lack of anatomical confirmation, some doubt exists in describing this relatively obscure anatomical structure (7). To our knowledge, this unnamed foramen is not yet termed in literature. During CT scan interpretation of the skull base, these less-known foramina can be mistaken for an abnormality in patients with neoplasia or vascular lesions (7). They can be easily overlooked if not incorporated into the interpreting radiologist’s search pattern, leading to delayed diagnosis, misinterpretation due to failure of recognition, and incomplete imaging of skull base entities (16).

## Abnormal Bony Outgrowths at the Skull Base

In a study on 100 dried adult human skulls, abnormal bony outgrowths and osseous structure around the foramen ovale were noted in 22% and 17% of skulls, respectively (1). The bony outgrowths were either protruding from the margins of the foramen ovale or from the cerebral surface of the greater wing of the sphenoid bone, obliterating and dividing the foramen ovale into two foramina. According to a study conducted by Berlis et al. (18), 12.5% of cases of foramen ovale were divided by osseous bridge and 0.8% of cases showed double foramen ovale (17). A study on 55 dried adult skulls and 20 sphenoid bones showed the presence of bony outgrowth surrounding the foramen spinosum and foramen ovale. It extended from the latter to the spine of the sphenoid. This could probably compress middle meningeal vessels resulting in neurogenic inflammation or cranial vasodilatation-induced migraine (18).

The greater wing of the human sphenoid, also forming the middle portion of the skull base consists of foramina such as foramen ovale, foramen spinosum and an inconstant symmetrical foramen of Vesalius (17, 19, 20). The foramen of Vesalius is found anteromedial to foramen ovale, its exocranial opening lies at the scaphoid fossa. When present, an emissary vein connecting the cavernous sinus with the pterygoid venous plexus traverses it. When absent, the emissary vein passes through the foramen ovale which is an alternate route of tumour invasion in the case of skull pathology. Nasopharyngeal tumours are also likely to invade the foramina following the course of tensor veli palatine muscle and Eustachian tube into the cranial cavity or vice versa to the skull base (20). The possibility of an emissary vein passing through the innominate foramina and causing potential tumour invasion or vascular lesion is cynical as it does not communicate with the cranial cavity. However, it is tempting to postulate that there could be a possibility of perineural spread of head and neck malignancies such as adenoid cystic carcinoma, squamous cell carcinoma, lymphoma, melanoma, basal cell carcinoma, adenocarcinoma, chondrosarcoma, a malignant mixed tumour which is usually associated with cranial nerve infiltration and skull base invasion occurring commonly along branches of trigeminal nerve passing through foramen ovale (21).

Further, meningiomas, neurolemmomas, cholesteatoma, nasopharyngeal tumours, vascular aneurysms, trauma, paranasal sinus infections and osteomyelitis are known to cause enlargement of skull base foramina. In contrast, fibrous dysplasia, Paget’s disease, osteoblastic metastasis and osteopetrosis cause foraminal narrowing leading to neural compression (22). We have presented anatomic relationships of the major foramina such as foramen ovale and foramen spinosum with that of an innominate foramen in an effort to point out additional foramina which may be clinically significant. There seems to be a possibility of involvement of the latter in pathological processes characterised by erosive or constrictive changes that can alter the size and shape of the foramina (22).

Vowing to the complexity of structures at the skull base, it is imperative that the skull base surgeons and radiologists are familiar with these newer entities at the skull base in localising skull base lesions, tumour staging, tumour extension and spread, and deciding the optimal surgical approach (22). Alternatively, avoiding injury and preservation of the branches of the mandibular nerve and lesser petrosal nerve, which may otherwise lead to paraesthesia in the area of distribution (9).

## Conclusion

In conclusion, though the incidence of clinically proven cases of compression due to these bony anomalies is limited, these unnamed foramina are definitely of interest, curiosity and confusion in terms of their development, rare location, and their interpretation.

## Figures and Tables

**Table 1:** Mean distance of innominate foramen from foramen ovale and foramen spinosum. Data expressed as mean, standard deviation (parenthesis) and range

Foramen	Side	Distance from foramen ovale (mm)	Distance from foramen spinosum (mm)
Innominate foramen	Right	1.26 (SD = 0.45) 0.8–2.0	4.7 (SD = 0.83) 4.0–6.0
	Left	1.43 (SD = 0.49) 1.1–2.0	5.8 (SD = 1.0) 4.8–6.7
